# Supplementary material for: BH3 Mimetic Sensitivity of Colorectal Cancer Cell Lines in Correlation with Molecular Features Identifies Predictors of Response
Source: Int J Mol Sci. 2021 Apr 7;22(8):3811. doi: 10.3390/ijms22083811 (PMC8067732; doi:10.3390/ijms22083811)
Supplement: Supplementary file 1 [file ijms-22-03811-s001.zip › ijms-1128665-supplementary.docx]

**Supplementary Figure S1.** Doses-Response curves of four BH3 mimetics and four combinations (1:1) in all CRC cell lines. Y axis indicates the relative cell viability normalized to untreated control.

**Supplementary Figure S2.** (A)IC50 range of four BH3 mimetics in single treatment, ****:p<0,0001, **:0,005<p<0,01; (B) Correlation analysis between sensitivity to ABT-263 and combined ABT-199 and A-1155463 in the CRC cell line panel. Spearman’s rank correlation was performed. Mann-Whitney test (two-tailed) was performed. (C) Flow cytometry of activation of caspase 3 induced by A-1155463+AZD5991 with or without 10uM Q-VD-OPh in HUTU-80; (D) Flow cytometry of PI incorporation induced by A-1155463+AZD5991 with or without 10uM Q-VD-OPh in HUTU-80;

**Supplementary Figure S3.** Correlation between target expression and sensitivity. (A-C) Correlation between IC50 of ABT-199(A), A-1155463(B) and AZD5991(C) to relative expression of BCL-2(A), BCL-XL(B) and MCL-1(C). Relative expression is normalized to 2,2,2-TCE signal as described. Spearman’s rank correlation was performed.

**Scheme 4.** Correlation analysis of IC50s of four combination (1:1) and molecular phenotypes in CRCs. (A) Microsatellite instability MSI, MSI microsatellite instable; MSS: microsatellite stable; (B) CpG Island Methylator Phenotype CIMP, CIMP-High: high CpG island Methylation; CIMP-Low: low CpG island Methylation; (C-G) Mutation: Mut: mutated; WT: wild type; *: p<0,05. Mann-Whitney (one-tailed) test was performed.
